# Latent Code Predictor for Accelerating Disparity Estimation in Stereo-Endoscopic Surface Reconstruction

**DOI:** 10.3390/s26082529

**Published:** 2026-04-20

**Authors:** Jiawei Dang, Bo Yang, Guan Yao, Chao Liu, Wenfeng Zheng

**Affiliations:** 1 School of Automation Engineering, University of Electronic Science and Technology of China, Chengdu 611731, Chinayaoguan1@huawei.com (G.Y.); 2 Department of Robotics, Laboratory of Computer Science, Robotics and Microelectronics of Montpellier (LIRMM), University of Montpellier, French National Center for Scientific Research (CNRS), 34095 Montpellier, France; liu@lirmm.fr

**Keywords:** latent vector prediction, generative model acceleration, disparity estimation, soft tissue reconstruction, endoscopic vision

## Abstract

Disparity estimation from stereo-endoscopic images is critical for 3D reconstruction in minimally invasive surgery (MIS). However, surgical environments have inherent interference factors including soft tissue deformation, motion blur, and photometric inconsistency. Currently, self-supervised generative networks such as StyleGAN offer an alternative method, but their reliance on iterative latent optimization leads to high computational latency and limits practical deployment. In this work, we propose a temporal latent prediction method to accelerate this optimization process. Instead of designing a brand new generator, our framework learns to predict an optimized initial latent vector, thereby reducing the number of optimization steps and per-frame inference time. Crucially, this prediction-guided mechanism does not alter the architecture or inference logic of the generator, ensuring the fidelity of reconstruction is comparable to that of the original method. Experiments on *Phantom* and *In vivo* datasets demonstrate that our method reduces average optimization steps by 16–59% and cuts per-frame latency by about 2.3×, compared to baseline predictors and initialization strategies. Importantly, the final photometric loss remains nearly identical across all methods, confirming that acceleration does not compromise reconstruction quality. These results position our approach as a practical step toward efficient, self-supervised stereo-endoscopic reconstruction in clinical settings.

## 1. Introduction

Efficient and chronological disparity estimation from stereo-endoscopic imagery plays a pivotal role in 3D reconstruction, surgical navigation, and scene understanding during minimally invasive surgery (MIS) [1,2,3]. Depth cues derived from sequential disparity maps enable safer surgical planning and continuous tool tracking. However, MIS imposes a series of challenges on disparity estimation, including frequent occlusions by surgical instruments, severe non-Lambertian reflections, soft tissue motion blur, and limited lighting conditions [4,5,6]. In addition, the scarcity of truth disparity datasets makes supervised learning impractical to deploy in the real world, due to the ethical and technical constraints.

### 1.1. Related Works

Traditional stereo matching techniques such as Thin-Plate Spline (TPS) interpolation, which rely on sparse feature correspondences and prior surface assumptions, have difficulty with complex and nonrigid anatomical structures [7,8,9]. With the advent of deep learning, end-to-end stereo matching networks have improved robustness. For example, AANet [10] and PSMNet [11] use deep cost volume encoding and spatial pyramid pooling (SPP) to extract features from images, achieving high accuracy in general scenarios. However, these supervised methods heavily depend on abundant labeled datasets, making them difficult to deploy in surgical scenarios.

Motivated by the critical need for accurate and efficient 3D reconstruction in surgery, recent research has explored a diverse range of strategies. To improve geometric reliability, Diaz-Ramirez et al. proposed a multi-ocular vision method with an adjustable baseline, demonstrating robust performance in 3D reconstruction tasks [12]. Addressing the critical need for efficiency, Cheng et al. developed a high-performance disparity prediction algorithm optimized for edge computing, effectively balancing accuracy with low latency [13]. Furthermore, Fan et al. introduced an unsupervised stereo matching scheme assisted by surface normal estimation, which successfully reduces ambiguity in texture-less regions [14]. The effectiveness of combining self-supervised depth networks with photometric constraints has been further validated by Recasens et al., who demonstrated that such pipelines can robustly handle tissue deformation and weak textures in monocular endoscopy [15]. Ji et al. integrated diffusion models with 3D Gaussian Splatting to simultaneously address motion blur and achieve real-time rendering in nasal endoscopy, highlighting the potential of generative priors in enhancing reconstruction quality [16]. Gil et al. focused on data quality enhancement, proposing an MAP estimation-based method to refine disparity maps for training lightweight neural network models, thereby improving overall depth perception accuracy [17]. Other works have also explored novel attention mechanisms and Transformer-based structures to capture long-context dependencies for better disparity prediction [18]. Although these studies have collectively driven technological advancements in the field of stereo vision, applying them directly to minimally invasive surgery remains challenging.

In this context, recent studies have introduced generative models, such as StyleGAN, into the field of disparity estimation. In this context, Yang et al. proposed a self-supervised model where a simplified StyleGAN generates disparity maps from compact latent vectors [19]. In their approach, the latent vector is iteratively optimized by using photometric reconstruction loss between the generated and real images. Although the initial generated disparity map may be inaccurate, multi-step optimization eventually converges to an optimal result. This method effectively captures complex anatomical structures without pixel-wise supervision.

Despite these strengths, a critical limitation persists that the latent vector optimization must be performed independently for each frame, requiring numerous iterations to converge. This results in high inference latency, hindering efficient deployment in latency-sensitive applications like intraoperative guidance. Moreover, processing frames independently ignores the temporal continuity inherent in endoscopic video streams.

### 1.2. Proposed Approach

Generative models without prediction guidance typically require a large number of inference steps to iterate from all-zero vectors to optimal vectors, like approaching each new end-point from a remote start-point for every frame. This iterative process is slow and inefficient in its early stages. However, if the generator starts near the target rather than from the origin, significant optimization time can be saved. Therefore, recognizing that the new target is constantly changing, a predictor can be designed to predict a start vector close to the target.

To achieve this approach, we propose a temporal latent prediction method built upon Yang’s framework. Rather than introducing an original generative model, our approach focuses on accelerating the existing optimization process. We introduce a predictive model in front of the generative model and propose a prediction-guided generative strategy. The predictive model leverages temporal information to predict high-quality initial latent vectors, which replace the all-zero initial latent vectors as inputs for the generative model.

Specifically, we integrate the trained feature mapping module and temporal predictor in front of the fixed StyleGAN3-based generator (we adopt the same method to train the StyleGAN-based generator following Yang’s work). We refer to this framework as PMG-Net, which consists of three functional components: (P): a temporal predictor for initial latent vectors; (M): a mapping net for extracting stereo image features; and (G): a generator for disparity synthesis. This framework combines the compressed information mapped from the current stereo images with historical reference information optimized by the generator to calculate the nonzero initial vector. The generator then rapidly optimizes this vector into an optimal vector, which is used both to synthesize the disparity map and as input for the predictor at the next frame.

Our design draws inspiration from the success of temporal prediction models in visual and physiological dynamics. While deep learning-based stereo matching methods have achieved significant progress [20,21,22]. Recurrent Neural Networks (RNNs), especially Gate Recurrent Unit (GRU) networks and Long Short-Term Memory (LSTM) networks, have been widely adopted due to their capacity to capture long-term dependencies [23]. Spatio-Temporal LSTM (ST-LSTM) networks have demonstrated robustness in the research of surgical vision, such as predicting heart motion and tracking tools, through integrating temporal evolution with spatial context [23]. Following these principles, we employ a GRU-based predictive module to enhance the efficiency of disparity estimation.

To effectively extract current frame features, we incorporate a feature extraction module ahead of the predictor. Inspired by AANet and PSMNet, this module utilizes spatial pyramid pooling (SPP) and cost aggregation to provide multi-scale representations, reducing errors in texture-less regions. Additionally, to ensuring computational efficiency while maintaining robustness [24,25], a refinement module comprising double ResNet layers [25] is added downstream for dimensionality reduction,. The refined features and the previous frame’s optimal latent vector jointly serve as inputs to the GRU network. The resulting high-quality initial latent vector guides the generator to achieve more precise and context-aware disparity estimation.

We adopt a self-supervised training strategy where the predictor is trained on labeled data generated from the generator. Specifically, for each frame, we obtain a disparity map and optimal latent vector using the well-trained generator. These pairs are organized into sequences, enabling the predictor to learn the mapping from visual features and historical states to optimized initial vectors. The mean squared error (MSE) loss is used to optimize the predictor.

The proposed method is trained and evaluated on two stereo-endoscopic datasets, a synthetic *Phantom* dataset and a real *In vivo* dataset, captured by the Da Vinci surgical robot. Experiments demonstrate that our strategy significantly reduces the number of optimization steps required per frame.

The main contributions of this work are summarized as follows:

- We propose a temporal latent prediction method that significantly reduces the optimization steps required for generative disparity estimation by providing an informed initial latent vector.
- We design a stereo feature encoding module and a GRU-based predictor to achieve more precise and computationally efficient initialization of the generator’s input vector.
- We validate our approach using a simplified StyleGAN3 generator, demonstrating that our acceleration strategy maintains reconstruction fidelity while improving efficiency compared to standard initialization baselines.

## 2. Materials and Methods

### 2.1. Experimental Setting

To validate the effectiveness and efficiency of our proposed method (referred to as PMG-Net), we conduct experiments on both synthetic and real-world stereo-endoscopic datasets.

This section details the experimental setting, datasets, implementation configuration, and evaluation indicators.

#### 2.1.1. Experimental Environment

All experiments are performed using a single high-performance GPU workstation. The environment settings are summarized in Table 1.

#### 2.1.2. Dataset

We evaluate PMG-Net on two open-source datasets provided by the Hamlyn Centre for Robotic Surgery, which are widely used in stereo-endoscopic disparity estimation research. The datasets include:

1. Real Heart Dataset derived from in vivo cardiac surgery [26,27].

This dataset was extracted from stereo video recordings of real heart surgery captured by the Da Vinci system. The original videos contain both left and right views such that a single frame has a total width of 720 pixels (360 pixels per view). Each frame is split into two 360×288 pixel views (left-view image and right-view image). A total of 1550 stereo frame pairs are extracted by frame-by-frame decomposition.

2. Silicone Heart Dataset created by a physical heart phantom [28].

The silicone dataset consists of two separate videos, one for the left view and one for the right view, each with 360×288 pixels of resolution and 1 min 37 s of duration. No splitting is required in this case. A total of 2425 stereo frame pairs are extracted directly from the two video streams. These synthetic data sequences simulate realistic camera motion and tissue texture and serve as a valuable substitute for testing domain adaptation.

**Figure 1:**
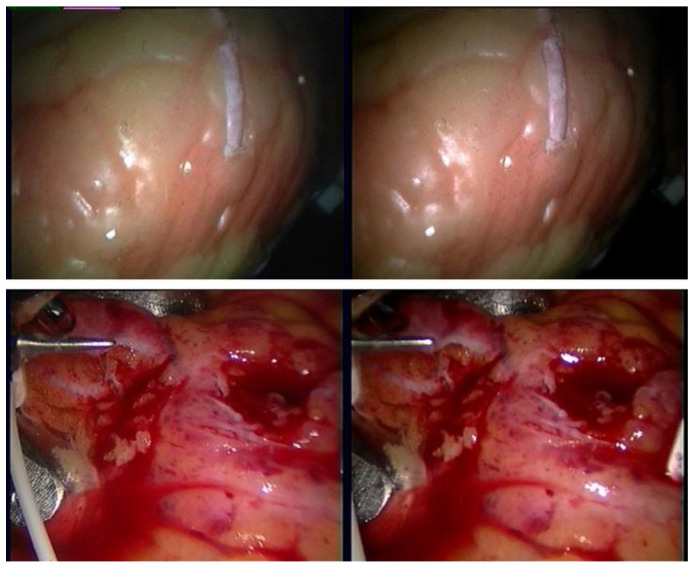
Stereo-endoscopic video frames of *Phantom* and *In vivo* datasets.

They are preprocessed in the same way as the real dataset for input of PMG-Net. A video screenshot of the original dataset is shown in Figure 1.

Both datasets were acquired using the Da Vinci surgical robot and are publicly available at: https://hamlyn.doc.ic.ac.uk/vision/ (accessed on 29 November 2025).

**Figure 2:**
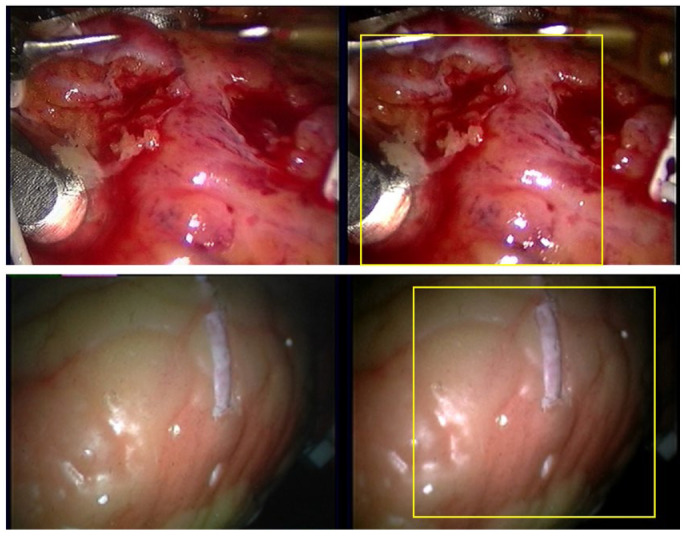
ROI (yellow rectangle) of Figure 1.

Since stereo-endoscopic images originate from point light sources, inconsistencies of bright and reflective areas both exist between the left and right views. Disparity values in these regions are invalid for appearance matching and gradient calculation. If disparity estimates from these areas are not skipped during loss calculation, the model may learn detrimental information, leading to wrong disparity estimates and partial surface collapses in reconstruction. Therefore, for both datasets, frame data are extracted at the native video frame rate (25 FPS), and the right views are cropped into 256 × 256 pixels of resolution as a ROI (Region of Interest), following prior work in [23], which are shown in Figure 2.

### 2.2. Methodology

In this section, we introduce the architecture and training methodology of PMG-Net.

#### 2.2.1. Predictor Network

**Figure 3:**
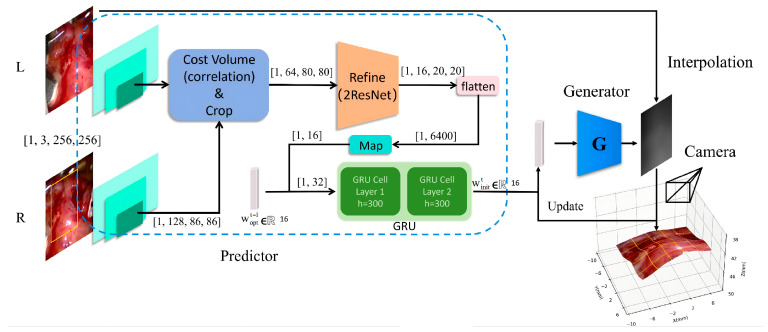
PMG-Net network structure. The yellow rectangles indicate the ROI on input stereo images. The blue dashed box encloses the GRU-based temporal predictor P, which combines current image features with the previous frame’s optimal latent vector ($w_{opt}^{t-1}$), to generate an informed initial latent vector ($w_{init}^{t}$). This vector initializes the generator G for iterative disparity optimization. The 3D camera symbol represents the virtual viewpoint reconstruction from the synthesized disparity map. Arrows indicate the data flow and the autoregressive feedback loop.

The predictor network consists of two parts, a stereo image mapping subnetwork and a latent vector predictive subnetwork. The former uses the SPP module, the cost aggregation module, the refinement module (double ResNet layers) and fully connected layers to perform feature extraction and feature aggregation. This design alleviates issues related to insufficient representation learning and high computational cost. Due to the low dimensionality of the data for autoregressive prediction, the latter uses a single-step GRU module with temporal context memory, combining the current image feature with the previous optimal latent vector, to ensure the predicted vector for the current disparity map aligns more closely with the generator’s final optimal vector. The network architecture diagram of PMG-Net is shown in Figure 3.

Compared to LSTM, the GRU simplifies the gate structure while maintaining consistent prediction performance. A well-trained generator can distribute the latent vector within a narrow generative space, effectively reflecting the mapping from the latent code vector to the image space. Our predictor learns the inverse mapping, embedding image information and historical latent states back into the latent space.

In practical applications, the predictor P requires only the current stereo-endoscopic image pair $\left(L^{t},R^{t}\right)$ and the previous optimal vector $w_{opt}^{t-1}$, obtained by the generator G, to obtain input in an autoregressive way and predict the latent vector mapping the current disparity. Since the dimension *N* of the stereo image encoding codes after dimensionality reduction is still much larger than dimension *K* of the optimal vector, the single-step GRU network further reduces the dimension of inputs. This process can be represented as $\left(K+N\right)\mapsto \;K$, as shown in Equation (1).

(1) $$w_{init}^{t}=P\left(\begin{array}{c} w_{opt}^{t-1}\\ M^{+}\left(L^{t}\right)\\ M^{+}\left(R^{t}\right) \end{array}\right)$$

where

- $w_{opt}^{t-1}$ is the optimal latent vector in the latent space $R_{W}^{K}\;from\;generator\;G$ at time t−1.
- L and R denote the left and the right images of the endoscope image pair.
- $M^{+}$ is an abstract function of the mapping subnetwork.
- $w_{init}^{t}$ represents the initial latent vector predicted by predictor P, which is also the input of the generator G at time t.

Obviously, no historical vector $w_{opt}^{t-1}$ exists for the first frame; a fixed zero-mean vector derived from the image domain serves as the starting point. Subsequent frames can be continuously trained or predicted based on the optimal latent vector from the previous moment. Empirical results show that the predictor stabilizes within 1–3 frames after the initial fixed-vector start, rendering the warm-up phase negligible for long-duration surgical videos.

#### 2.2.2. Loss Function of Training

The training of predictor P is formulated as a regression task optimized using mean squared error (MSE) loss, which is shown in Equation (2).

(2) $${loss}_{MSE}=\frac{1}{N}\sum_{i=1}^{K}(w_{i}-{\hat{w}}_{i}{)}^{2}$$

where K is the dimensionality of the latent space.

A lower MSE indicates that the predicted vector is closer to the generator’s optimal input, reflecting superior mapping, encoding and prediction capability. It is important to clarify that while MSE evaluates the quality of the initialization, it does not directly measure the final reconstruction quality.

#### 2.2.3. Algorithm Description

The proposed strategy consists of three stages, offline generator training, offline predictor training, and online inference, as summarized in Algorithm 1.

For clarity, we utilize the subscripts of latent vectors to indicate their role (e.g., $w_{opt}$ means the final optimal vector, $w_{init}$ means the initialization latent vector from predictor P, and $w_{train}$ means the optimal vector optimized independently by the generator G, serving as the label for the P), and we also utilize the superscripts of latent vectors for temporal indexing (e.g., $w^{t}$ means the current time-step and $w^{t-1}$ means the last time-step).

**Algorithm 1.** Prediction-Guided Generative Model FlowInput: Left-view image $I_{l}$, right-view image $I_{r}$, latent vector $w_{opt}^{t-1}$.Output: Reconstructed soft tissue surface Surf.Training stage:
1.Train the generator G following Yang’s work.

2.Generate dataset tuples $\left(I_{l},I_{r},w_{train}\right)$ using G.

3.Fix G.

4.Train predictors $\left(P_{SiamG},\;P_{MG},\;P_{PMG}\right)$ on the generated dataset.
Inference stage:
1.P predicts the initial latent code: $w_{init}^{t}=P\left(I_{l},I_{r},w_{opt}^{t-1}\right)$.

2.Initialize latent code: $w=w_{init}^{t}.$

3.Reconstruct right image: ${\hat{I}}_{r}=Interp\left(G\left(w\right),I_{l}\right).$

4.While photometric loss $L_{pho}\left({\hat{I}}_{r},I_{r}\right)>\epsilon$, perform:

5.Compute total loss: $L=\frac{\lambda }{N}\left.|G\left(w\right)-I_{r}\right.{|}_{2}^{2}+L_{pho}\left(\hat{I_{r}},I_{r}\right)$.

6.      Update latent code: $w=w-\eta F\left(\nabla_{w}L\right)$.

7.      Reconstruct right image again: ${\hat{I}}_{r}=Interp\left(G\left(w\right),I_{l}\right).$

8.End While

9.Storage optimal latent code: $w_{opt}^{t}=w.$

10.Compute depth map: $Depth=\frac{bf}{G\left(w\right)}$, where b is the baseline of the stereo camera and f is the focal length of the stereo camera.

11.Render final surface: $Surf=Render\left(Depth,I_{r}\right)$


The central idea of this algorithm is to build existing research upon the previous work, which trained a generator G for high-fidelity image synthesis. We additionally train a predictor P to estimate initial latent vector $w_{init}^{t}$ as a starting point, thereby reducing the number of steps required for the G to get $w_{opt}^{t}$.

Stage 1: Offline Generator Training

Following Yang [19], we train the StyleGAN3-based generator G to map latent codes to disparity maps. Once trained, G remains fixed in all subsequent steps.

Stage 2: Offline Predictor Training

With the well-trained G, we generate supervision data ($I_{l},\;I_{r},\;w_{train}$) to train three predictors.

While all predictors use the Adam optimizer with weight_decay = 0, their architectures and training configurations differ due to functional design:

- SiamG-Net and MG-Net: Feed-forward models that process each frame independently. They are trained with a batch size = 5, a learning rate = $1\;\times 10^{-4}$, and a shuffled dataset.
- PMG-Net: Incorporates a GRU-based temporal module. Due to this sequential dependency, it is trained with a batch size = 1, no dataset shuffling, and a slight higher learning rate = $1\times 10^{-3}$, to stabilize gradient updates.
- All models are trained on the same dataset and validated under identical metrics.

Stage 3: Online Inference

At inference, P produces an initial latent vector $w_{init}^{t}=$ $P\left(I_{l},\;I_{r},\;w_{opt}^{t-1}\right)$, which is then fed into G to synthesize a disparity map, from which a right-view image ${\hat{I}}_{r}$ is reconstructed. If the photometric loss between ${\hat{I}}_{r}$ and the real $I_{r}$ exceeds threshold ϵ, the latent vector is iteratively optimized using gradient descent, where the Adam optimizer uses a fixed learning rate of $5\times 10^{-3}$ and runs for up to 150 iterations. Once converged, the final depth map is computed and the 3D surface is reconstructed from Depth and $I_{r}.$

This hybrid approach, which combines offline training with online optimization, achieves rapid initialization through the predictor P. Compared to purely online generation using a generator G alone, it enhances reconstruction efficiency while maintaining nearly identical accuracy.

### 2.3. Evaluation Metrics

We adopt specific indicators to comprehensively evaluate the effectiveness of the latent code predictor P in the proposed model, distinguishing between metrics that measure optimization efficiency and those that assess reconstruction fidelity.

The first indicator is the mean squared error (MSE) between the predicted initial latent code $w_{init}^{t}$ and the final optimal latent code $w_{opt}^{t}$. It is important to clarify that MSE serves as an efficiency metric rather than a direct measure of visual quality; it quantifies the predictor’s capacity to map image features into the latent space and provide a starting point close to the convergence target. A lower MSE suggests that P provides a better starting point for convergence and significantly reduces the search space for the generator. For baseline models without temporal predictive capabilities, MSE also provides a reference for the quality of their static latent encoding.

The second indicator is the number of optimization steps required by the generator to reach a stable photometric loss when initialized with $w_{init}^{t}$. Fewer steps directly correlate with faster convergence and reduced per-frame latency, evaluating how effectively the predicted vector guides the generator toward the optimal solution. Under similar final loss levels, a smaller number of required steps indicates superior initialization ability and helps mitigate local overfitting during the iterative process.

Crucially, to ensure that acceleration does not compromise output quality, we utilize the final photometric loss as the primary fidelity metric. This value measures the pixel-wise consistency between the reconstructed image and the real target after convergence. Our results demonstrate that the final photometric loss remains nearly identical across all methods, confirming that the speed improvements brought by P do not come at the cost of reconstruction fidelity. In other words, MSE and iteration count reflect the efficiency gains of our approach, and the consistent photometric loss validates that the predictor successfully guides the generator to the same high-quality solution more rapidly. Therefore, these metrics together provide a holistic assessment of both the latent prediction performance and the practical benefit of integrating temporal prediction into the generative pipeline.

## 3. Experimental Results and Analysis

### 3.1. Validation Loss During Training

**Figure 4:**
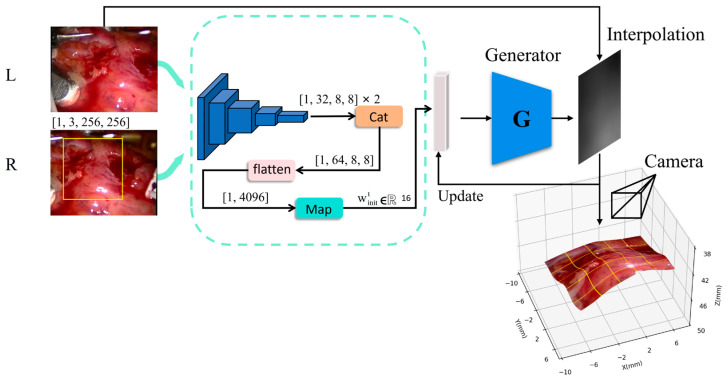
SiamG-Net network structure. This model employs the Siamese network to extract features from left and right images, which are then aggregated and directly mapped to an initial latent vector without temporal information. This architecture serves as a baseline to evaluate the contribution of temporal prediction. Components not re-described in Figure 4 follow the same configuration as those in Figure 3.

**Figure 5:**
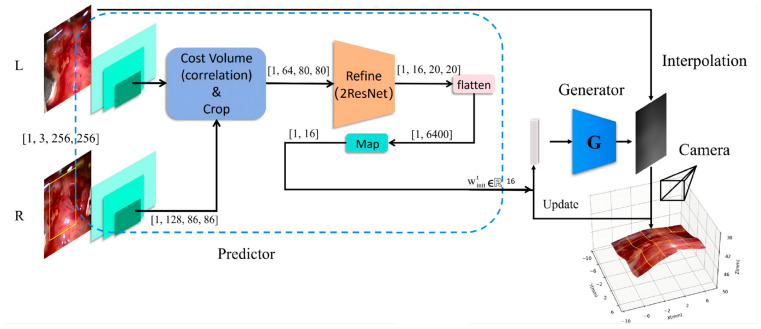
MG-Net network structure. This model retains the same feature mapping subnetwork as PMG-Net but removes the GRU-based module (in the blue dashed box). The mapped feature vector is directly used as the initial latent vector ($w_{init}^{t}$) for the G, ignoring frame-to-frame continuity. Comparing MG-Net with PMG-Net isolates the performance gain from the temporal latent prediction strategy. Components not re-described in Figure 5 follow the same configuration as those in Figure 3.

To validate the contribution of the predictor P within the prediction-guided generative framework, we introduce two image mapping networks, SiamG-Net and MG-Net, for comparative verification, which are shown in Figure 4 and Figure 5. The former is inspired by Siamese-Net [29], a Siamese matching network with ResNet for feature extraction, which has demonstrated strong capabilities in feature extraction from similar image pairs. MG-Net retains the mapping subnetwork and generator of our proposed method but removes the temporal predictive subnetwork. Consequently, both baseline models extract low-dimensional feature vectors from stereo images on a frame-by-frame basis, lacking temporal awareness. In contrast, PMG-Net integrates both the mapping subnetwork and the GRU-based predictive subnetwork to leverage temporal coherence.

In addition to the network models for comparative verification, two initialization methods for the latent vector are selected as benchmarks: $w_{opt}^{t-1}$ and ${\bar{w}}_{opt}^{train}$. $w_{opt}^{t-1}$ represents a method that always adopts the optimal latent code vector of the previous frame from the generator. Theoretically, the code vectors of the adjoining frame are spatially analogous, so that using the continuous frame predictive method effectively reduces the number of iterations, similarly to the benchmark in the historical prediction process. ${\bar{w}}_{opt}^{train}$ represents a method that always adopts the center position of all $w_{opt}^{train}$ in the training data as the starting point. Searching for the optimal vector based on the center position of the historical optimal vectors can also reduce the number of iterations, similarly to the benchmark in the mean prediction process.

**Figure 6:**
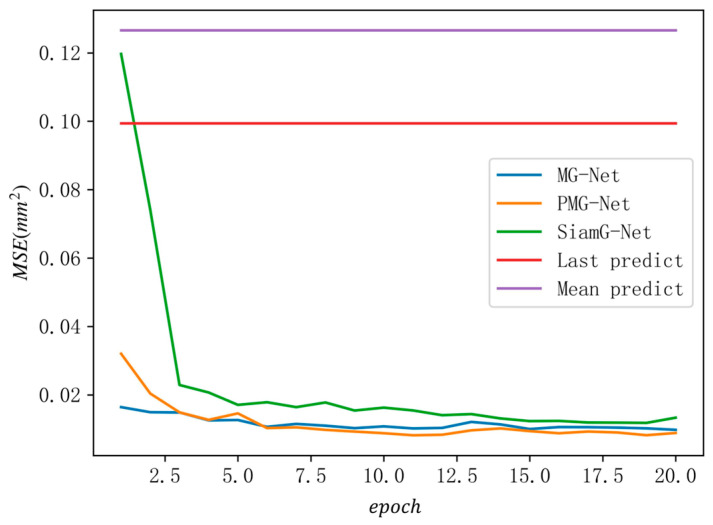
Validation loss of each model during training on the *Phantom* dataset.

As shown in Figure 6, all three models demonstrate significant learning capabilities on the *Phantom* dataset, with MSE scores significantly lower than the two initialization methods, based on the last historical prediction and the mean historical prediction.

In the early stages of training, MG-Net exhibited superior prediction capabilities. PMG-Net’s GRU module had not yet learned the data patterns, which implies that its mapping performance was weaker than that of MG-Net. Limited by the network structure, SiamG-Net’s prediction performance was even weaker than the last historical prediction. As training epochs increased, SiamG-Net began to learn the features of the stereo images, and its MSE significantly decreased, but it was still weaker than MG-Net. At same time, PMG-Net’s GRU module had already learned the historical motion patterns of the latent code vector. With the participation of the mapping subnetwork, it achieved a prediction task superior to that of the other two models.

### 3.2. Computational Efficiency Test

This section compares the models (MG-Net, SiamG-Net, and PMGNet). The test results on the *In vivo* and *Phantom* datasets are shown in Table 2. Bold values indicate the best performance.

The single-frame time consumption of the prediction-guided generative model in the application phase is calculated as shown in Equation (3).

(3) $$t_{all}=t_{p}+N_{step}*t_{g}$$

where $t_{p}$ represents the prediction duration, step represents the number of iterations, and $t_{g}$ represents the single generation time.

Results show that all three learned models achieve fewer steps to reach optimal vector and optimal disparity than the two static initialization baseline methods. However, the number of iterations required varies significantly between different starting points, likely due to the higher inter-frame continuity instead of the spatial cohesion of the latent vector. And the high variance in latent vectors also leads to significant differences between methods of different starting points.

**Figure 7:**
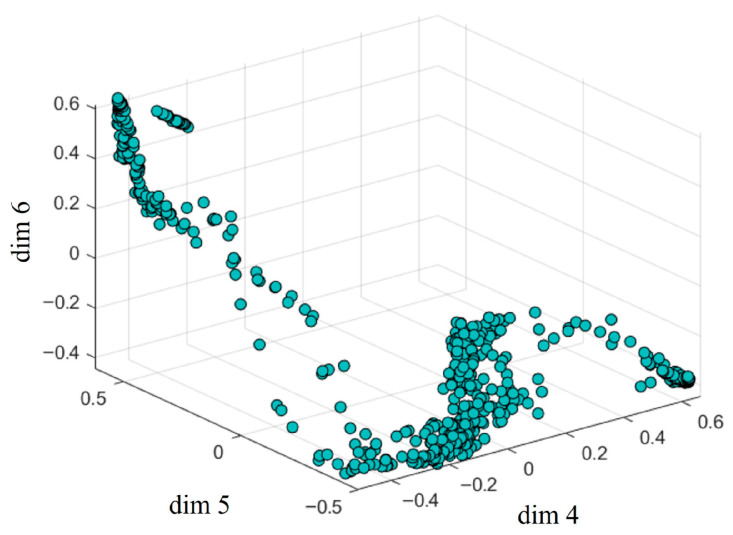
Sampling $w_{train}$ low-dimensional distribution.

As illustrated in Figure 7, the learned latent codes do not follow a uniform distribution, but instead exhibit a clustered multi-modal structure; most samples concentrate in several well-separated dense regions, while few lie in between. These clusters correspond to characteristic soft tissue deformation states observed during surgery, for example relaxed, contracted, and torsional configurations. Consecutive frames tend to remain within the same cluster for multiple time-steps, and transitions between clusters occur gradually and predictably. This temporal coherence validates that modeling latent dynamics is meaningful and feasible. It also explains why our GRU-based predictor, which captures such temporal patterns, outperforms static initialization strategies.

Experimental results confirm that by leveraging both current stereo features and historical latent states, our predictor enables the generator to initialize its search at a position much closer to the final optimum. Table 2 shows that PMG-Net achieves the lowest per-frame latency among all compared methods, with an average inference time of 0.33–0.37 s on our current workstation setup. This represents a 2.3× speedup over the baseline initialization strategies.

While the current implementation yields approximately 3 FPS, which falls short of the strict 25–30 FPS requirement for fluid video-rate navigation, our results demonstrate a clear pathway toward real-time performance. According to Equation (3), the total latency is dominated by the iterative term ($N_{step}*t_{g}$). Our method reduces $N_{step}$ by 50–59% compared to the baselines. Since the generator operation ($t_{g}$) is highly parallelizable and amenable to hardware acceleration (e.g., via TensorRT optimization or deployment on embedded GPUs), improvement in single-step throughput is sufficient to achieve the target of ≥25 FPS. Crucially, such hardware-level optimizations would yield diminishing returns for the baseline methods due to their high iteration count, whereas our reduced step count makes the real-time goal algorithmically feasible and engineeringly realistic. Thus, PMG-Net provides the necessary algorithmic foundation for future high-speed clinical deployment.

## 4. Discussion

The experimental results demonstrate that our proposed temporal latent prediction method (PMG-Net) effectively combines temporal latent prediction with spatial stereo encoding to improve the efficiency of latent optimization in minimally invasive surgical scenarios. Compared to baseline models including MG-Net and SiamG-Net, PMG-Net exhibits fewer optimization steps, by 33–59% on *Phantom* and 16–45% on *In vivo*, respectively. By leveraging both image features and historical latent codes, the predictor provides the generator with an initial vector closer to the optimal vector, accelerating convergence and reducing the risk of local overfitting. Crucially, as defined in our metric analysis, this acceleration serves as an efficiency gain without compromising reconstruction fidelity. The final photometric loss remains nearly identical across all methods. It is confirmed that PMG-Net enables faster convergence to the same high-quality solution, rather than trading off accuracy for speed. Although explicit jitter metrics were not computed, the recurrent nature of the GRU inherently imposes temporal smoothing constraints.

In terms of computational performance, PMG-Net achieves the shortest per-frame processing time among all compared methods, with an average latency of approximately 0.4 s. While this current speed does not yet meet the strict 25–30 FPS standard for fluid video-rate navigation, it represents a critical 2.3× speedup over static initialization strategies. As analyzed in Section 3.2, this substantial reduction in algorithmic complexity (iteration count) lays a solid foundation for future real-time deployment on optimized hardware. Furthermore, the model demonstrates robust prediction capabilities across both synthetic (silicone heart) and real (in vivo heart) datasets. This consistent performance across domains with different tissue properties and lighting conditions suggests that the learned temporal patterns are generalizable within the surgical context, addressing concerns about domain shift.

However, certain limitations remain. First, while the predictor considers the temporal coherence, it is still constrained by the variability and discontinuity of latent space distributions. The distribution gap in the class, observed in the experiments, highlights the need for more complex temporal modeling and generalization ability. Second, although MSE and iteration counts effectively measure efficiency and photometric loss validates fidelity, future work could integrate task-specific clinical metrics (e.g., surface smoothness or instrument tracking accuracy) to further validate clinical applicability. Finally, achieving the target of ≥25 FPS remains an engineering milestone that will benefit from dedicated inference acceleration techniques, such as model quantization and cross-platform deployment studies.

It is important to note that our framework is designed for scene-specific adaptation rather than direct zero-shot cross-dataset generalization. In clinical practice, a short calibration sequence at the beginning of a procedure can be used to adapt the predictor online, making it robust to variations in lighting, tissue appearance, and camera setup specific to each patient.

## 5. Conclusions

This article presents a temporal latent prediction method (referred to as PMG-Net) for accelerating self-supervised disparity estimation in stereo endoscopy. By introducing a GRU-based predictor that integrates stereo image features with historical latent coding vectors, our approach provides high-quality initial vectors for the generative model, significantly reducing the required optimization steps while maintaining reconstruction accuracy. Experimental evaluations on open-source surgical datasets demonstrate that our method outperforms baseline initialization strategies in both computational efficiency and temporal stability.

The findings confirm that incorporating temporal prediction into generative optimization loops offers a promising solution for efficient and adaptive disparity estimation under challenging surgical conditions. Unlike purely self-supervised generation methods, our approach exploits the inherent continuity of endoscopic video to minimize redundant computations. In future work, we aim to extend the predictor to more advanced temporal architectures and explore its deployment on embedded surgical platforms, paving the way for robust, low-latency 3D reconstruction and navigation in minimally invasive surgery.

## Figures and Tables

**Table 1:** Experimental environment and implementation configuration.

Category	Configuration
GPU	NVIDIA RTX 3090 (24 GB)
CPU	Intel Core i9-12900K
Memory	128 GB DDR5
Operating System	Ubuntu 20.04 LTS
Deep Learning Framework	PyTorch 1.13.0
CUDA Version	11.6
Latent Dimension	64
Generator Type	StyleGAN3
Optimizer	Adam $\left(\beta_{1}=0.9,\beta_{2}=0.999\right)$
Learning Rate	$1\times 10^{-4}$
Batch Size	8
Image Resolution	256×256 (after cropping)
Optimization Steps	10 per frame

**Table 2:** Time statistics of prediction-guided generative model on *In vivo*/*Phantom* dataset.

Method	$loss_{pho}\left(pixel^{2}\right)↓$	$Iterations\left(Times\right)↓$	$Singleframetime\left(s\right)↓$
Mean optimal vector (${\bar{w}}_{train}$)	63.50888/41.47058	23.35/25.27	0.7007/0.7583
Last optimal vector ($w_{opt}^{t-1})$	63.44433/41.55592	20.73/31.68	0.6229/0.9514
SiamG-Net (Siamese-Net)	62.61963/42.29782	15.38/18.89	0.4664/0.5717
MG-Net (Mapping)	62.48380/41.78096	12.34/15.75	0.3902/0.4925
PMG-Net (Mapping + GRU)	62.52708/41.80012	10.63/11.86	0.3379/0.3748

## Data Availability

The dataset analyzed in this study is publicly available. The *In vivo* and *Phantom* datasets can be accessed through https://hamlyn.doc.ic.ac.uk/vision/ (accessed on 29 November 2025).
